# Multipolarized radar reveals shallow subsurface structure and middle-late Amazonian aqueous activity in Utopia Planitia, Mars

**DOI:** 10.1093/nsr/nwaf505

**Published:** 2025-11-14

**Authors:** Yike Liu, Tianfan Yan, Xiaoguang Qin, Ling Chen, Jinhai Zhang, Yang Liu, Yangting Lin, Fuyuan Wu, Ross N Mitchell, Zhendong Zhang, Jiangjie Zhang, Haiwei Wang, Chao Li, Yibo Wang, Bin He, Yikang Zheng, Lei Zhang, Juan Li, Kaichang Di, Wenhui Wan, Honglei Lin, Jiangang Wang, Jinlai Hao, Xin Wang, Pan Zhao, Xu Wang, Yongxin Pan

**Affiliations:** Key Laboratory of Deep Petroleum Intelligent Exploration and Development, Institute of Geology and Geophysics, Chinese Academy of Sciences, Beijing 100029, China; Key Laboratory of Deep Petroleum Intelligent Exploration and Development, Institute of Geology and Geophysics, Chinese Academy of Sciences, Beijing 100029, China; State Key Laboratory of Lithospheric and Environmental Coevolution, Institute of Geology and Geophysics, Chinese Academy of Sciences, Beijing 100029, China; State Key Laboratory of Lithospheric and Environmental Coevolution, Institute of Geology and Geophysics, Chinese Academy of Sciences, Beijing 100029, China; Key Laboratory of Earth and Planetary Physics, Institute of Geology and Geophysics, Chinese Academy of Sciences, Beijing 100029, China; State Key Laboratory of Space Weather, National Space Science Center, Chinese Academy of Sciences, Beijing 100190, China; Key Laboratory of Earth and Planetary Physics, Institute of Geology and Geophysics, Chinese Academy of Sciences, Beijing 100029, China; State Key Laboratory of Lithospheric and Environmental Coevolution, Institute of Geology and Geophysics, Chinese Academy of Sciences, Beijing 100029, China; State Key Laboratory of Lithospheric and Environmental Coevolution, Institute of Geology and Geophysics, Chinese Academy of Sciences, Beijing 100029, China; Key Laboratory of Earth and Planetary Physics, Institute of Geology and Geophysics, Chinese Academy of Sciences, Beijing 100029, China; Key Laboratory of Deep Petroleum Intelligent Exploration and Development, Institute of Geology and Geophysics, Chinese Academy of Sciences, Beijing 100029, China; Key Laboratory of Deep Petroleum Intelligent Exploration and Development, Institute of Geology and Geophysics, Chinese Academy of Sciences, Beijing 100029, China; Key Laboratory of Deep Petroleum Intelligent Exploration and Development, Institute of Geology and Geophysics, Chinese Academy of Sciences, Beijing 100029, China; Key Laboratory of Earth and Planetary Physics, Institute of Geology and Geophysics, Chinese Academy of Sciences, Beijing 100029, China; Key Laboratory of Deep Petroleum Intelligent Exploration and Development, Institute of Geology and Geophysics, Chinese Academy of Sciences, Beijing 100029, China; Key Laboratory of Deep Petroleum Intelligent Exploration and Development, Institute of Geology and Geophysics, Chinese Academy of Sciences, Beijing 100029, China; Key Laboratory of Deep Petroleum Intelligent Exploration and Development, Institute of Geology and Geophysics, Chinese Academy of Sciences, Beijing 100029, China; Key Laboratory of Earth and Planetary Physics, Institute of Geology and Geophysics, Chinese Academy of Sciences, Beijing 100029, China; Key Laboratory of Earth and Planetary Physics, Institute of Geology and Geophysics, Chinese Academy of Sciences, Beijing 100029, China; State Key Laboratory of Remote Sensing Science, Aerospace Information Research Institute, Chinese Academy of Sciences, Beijing 100101, China; State Key Laboratory of Remote Sensing Science, Aerospace Information Research Institute, Chinese Academy of Sciences, Beijing 100101, China; Key Laboratory of Earth and Planetary Physics, Institute of Geology and Geophysics, Chinese Academy of Sciences, Beijing 100029, China; Key Laboratory of Earth and Planetary Physics, Institute of Geology and Geophysics, Chinese Academy of Sciences, Beijing 100029, China; State Key Laboratory of Lithospheric and Environmental Coevolution, Institute of Geology and Geophysics, Chinese Academy of Sciences, Beijing 100029, China; State Key Laboratory of Lithospheric and Environmental Coevolution, Institute of Geology and Geophysics, Chinese Academy of Sciences, Beijing 100029, China; Key Laboratory of Earth and Planetary Physics, Institute of Geology and Geophysics, Chinese Academy of Sciences, Beijing 100029, China; State Key Laboratory of Lithospheric and Environmental Coevolution, Institute of Geology and Geophysics, Chinese Academy of Sciences, Beijing 100029, China; Key Laboratory of Earth and Planetary Physics, Institute of Geology and Geophysics, Chinese Academy of Sciences, Beijing 100029, China; College of Earth and Planetary Sciences, University of Chinese Academy of Sciences, Beijing 100049, China

**Keywords:** Zhurong rover, quad-polarized ground-penetrating radar, radar imaging, Utopia Planitia on Mars, aqueous activity

## Abstract

The Martian subsurface preserves a record of the geological and climatic evolution of Mars beyond that exposed at the surface. The Zhurong rover of the Tianwen-1 mission landed in southern Utopia Planitia on Mars and conducted a high-frequency quad-polarized ground-penetrating radar survey. High-frequency radar images with ∼5 cm vertical resolution in the immediate subsurface reveal three distinct subsurface layers, buried craters, centimeter-scale layered sediments, and widespread northward-sloping features, some of which likely formed in aquatic environments during the middle to late Amazonian period. These findings suggest that aqueous activity in Utopia Planitia persisted throughout this period, extending the known timeline of water-related processes on Mars and providing new insights into the red planet’s geological and climatic evolution.

## Teaser

High-resolution subsurface radar data from the Zhurong rover reveal fine-scale stratified sediments, buried craters, and northward-dipping features indicative of aqueous processes in southern Utopia Planitia during the middle-to-late Amazonian (~750 Ma). These observations provide some of the youngest low-latitude evidence for water activity on Mars and extend the timeline of known aqueous processes, offering fresh insight into the planet’s geological and climatic evolution.

## INTRODUCTION

**Figure 1.**
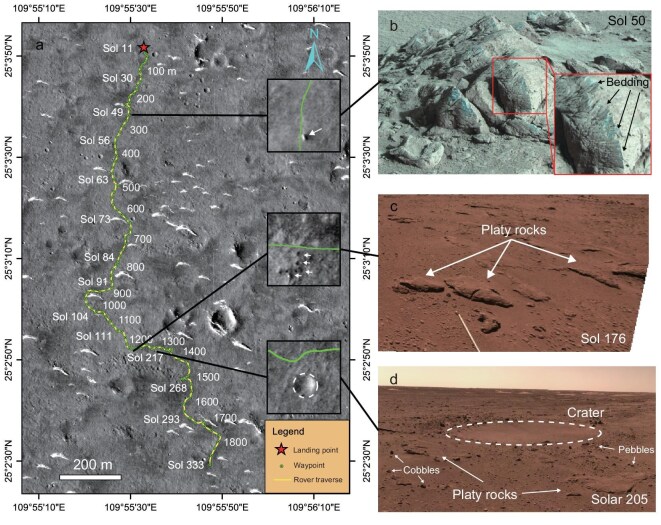
Map showing the location and traverse of the Zhurong rover. (a) Path of the Zhurong rover traversing Utopia Planitia of Mars. The base map image was taken by the Tianwen-1 orbiter high-resolution imaging camera (HiRIC). The three inset images in (a) were taken by NASA HiRISE (Image: NASA/JPL/University of Arizona), showing a bird’s-eye view of rocky blocks, platy rocks, and craters. The Zhurong rover moved 1.9 km in 323 sols (from Sols 11–333) and took photographs of geomorphic features, including platy rocks, bedding laminations, ejecta, craters, and boulders, via the Multispectral Spectral Camera (MSCam) (b) and NaTeCam (c, d).

The aim of the Tianwen-1 mission is to explore the geomorphic features, subsurface composition, and structure of the southern Utopia Planitia *in situ* to investigate potential aqueous activity in the northern plain of Mars [1–3]. Equipped with six scientific payloads (Fig. S1a), including the Rover Penetrating Radar (RoPeR), the Navigation and Terrain Camera (NaTeCam), the Mars Surface Composition Detector (MarSCoDe), and the Multispectral Camera (MSCam), the Zhurong rover landed on 15 May 2021, in the southern Utopian Planitia of Mars (25.066°N, 109.925°E) (Fig. 1). Utopia Planitia, the vast plain in the northern hemisphere of Mars [4–8], was flooded and filled by Hesperian-aged Vastitas Borealis Formation (VBF) materials [9,10]. Morphological features support the hypothesis that the surface layer in the VBF contains a significant amount of volatiles [11]. Since the landing of the Zhurong rover, numerous studies have identified a variety of surface morphological features, including landforms, craters, pebbles, polygonal, small dunes, and platy rocks, providing detailed characterizations of the regional topography [12–15]. Recent surface investigations using the Zhurong rover’s instruments, including MarSCoDe, NaTeCam, and MSCam, have revealed abundant hydrated sulfate- and silica-bearing materials. The hydrated materials have been observed on platy rocks, duricrusts, aquatic-associated cracks, and bedding structures [16–18]. Additionally, one study identified surface crusts, cracks, aggregates, and bright polygonal ridges associated with hydrated and salt-rich materials [17].

While orbital radar-sounding surveys have revealed abundant water-ice volumes in western Utopia Planitia [19,20], the shallow subsurface structure and composition of southern Utopia Planitia using a rover *in situ* have not been thoroughly explored. The Zhurong rover has traveled 1.9 km south since landing in southern Utopia Planitia. Unlike the other five payloads on the rover designed to detect surface features, the RoPeR instrument is the only payload capable of exploring shallow subsurface structures to depths of tens of meters. The low-frequency (15–95 MHz) single-channel data from RoPeR-Ch1 were used to identify a multilayer structure along a 1.1 km traverse of the Zhurong rover in southern Utopia Planitia [21]. Low-frequency radar data reveal that the landing site area has experienced two periods of sediment deposition [21]. The first occurred between 3.5 and 3.2 billion years ago, during the late Hesperian to early Amazonian period, when a catastrophic flood occurred. Sediments from this time are found ∼30 to 80 m below the surface. The second resurfacing began ∼1.6 billion years ago and was driven by a smaller flood [14,22]. These floods, likely triggered by obliquity changes on Mars, deposited sediments at depths of 10–30 m [14,21], where buried polygonal terrain and an ancient coastline were identified using low-frequency data [23,24]. Crater counting statistics from recent research at the Zhurong landing site suggest that the materials in the immediate subsurface may be as young as ∼757 Ma [25]. This implies that the region likely underwent a resurfacing event during the middle to late Amazonian period.

However, this low-frequency channel is unable to resolve near-surface structures and geological features at depths of less than 10 m due to strong signal noise and wavelength constraints. Understanding these near-surface characteristics is crucial for revealing geological and climatic evolution during the middle to late Amazonian. In addition to the low-frequency channel RoPeR-Ch1, the Zhurong rover, for the first time on Mars, deployed high-frequency (450–2150 MHz) quad-polarization radar antennas (RoPeR-Ch2) (Fig. S1b). These antennas are composed of co-polarized HH (horizontal transmission and horizontal reception) and VV (vertical transmission and vertical reception), as well as cross-polarized HV (horizontal transmission and vertical reception) and VH (vertical transmission and horizontal reception) components. In this work, we analyzed data from the RoPeR-Ch2 along a 1.9 km traverse (Sols 11 to 333) and obtained high-resolution images of the shallowest structures with a vertical resolution of 5 cm.

## RESULTS

### Shallow subsurface structures revealed by high-frequency multichannel radar

**Figure 2.**
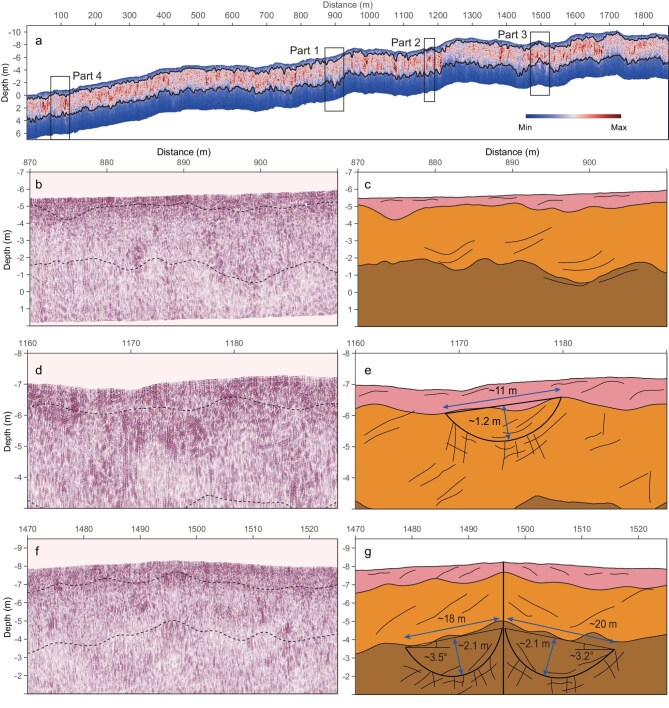
Profiles of high-frequency radar data from HV imaging. The data were acquired by the Zhurong rover from Sols 11–333 along a 1.9 km traverse. (a) Meter-scale HV reflectivity image displayed without automatic gain control (AGC), revealing three layers down to a depth of 7 m, where the top black line indicates the top layer, with an average depth of 0.65 m, ranging from 0.47 to 0.83 m deep, and the second line shows the bottom of the middle layer, with an average depth of 3.93 m, ranging from 3.65 to 4.21 m deep (Methods and Fig. S3). The two black lines were calculated via an automatic statistical zonation algorithm (see Methods); (b), (d), and (f) (with AGC) magnified selections of HV images of profiles 1, 2, and 3, while profile 4 is depicted in Fig. 3. The corresponding geological interpretation maps shown in (c), (e), and (g) exhibit a concave feature, that is, one buried crater in the middle layer, and another buried crater in the bottom layer (which appears as two craters because the rover took a U-turn and imaged the same crater twice.

The RoPeR-Ch2 data, after data processing (Methods and Fig. S2), reveal that the immediate subsurface of the landing area comprises three layers, which extend down to a depth of 7 m according to the HV channel (Fig. 2a). The three layers exhibit weak (top), strong (middle), and weak (bottom) reflectivity characteristics (Fig. 2a, Methods, and Fig. S3). No sharp interfaces are identified in the subsurface, suggesting that the immediate subsurface contains rock blocks and clastic deposits. The lower boundary of the top layer has a depth range of ∼0.47–0.83 m (average depth of 0.65 m; see Methods and Fig. S3). A retro rocket that carried the Zhurong rover and lander during landing excavated a 0.4-m-deep hole when the Zhurong lander touched down [26,27]. The exposed subsurface material in and around this hole consisted mainly of layered, unconsolidated gravel and sandy sediments [27]. The middle layer’s lower boundary, ranging from 3.65–4.21 m (average depth of 3.93 m), exhibits strong reflectivity (Fig. 2a and Fig. S3). The lower boundary of the bottom layer, which extends to depths of 7 m or more, exhibits weaker reflectivity than the overlying middle layer.

The NaTeCam and the High-Resolution Imaging Orbital Camera (HiRIOC) [28] of the Tianwen-1 mission identified numerous visible craters on the surface (Fig. 1a) [26–29] around the Zhurong landing site. The Zhurong rover’s high-frequency radar profiles have also revealed concave and bowl-shaped features interpreted as buried craters (Fig. 2d–g). These bowl-shaped features are visible in the imagery of the subsurface at the Zhurong landing site. One of these buried craters, located at a depth of 1.9 m, situated between 1168–1180 m along the traverse (Fig. 2d and e), has a diameter of 11 m and a crater thickness of 1.2 m. This buried crater is contained within the middle layer and is covered by a 0.7 m-thick top layer. In addition, two deeper craters within the lower layer are found next to each other from 1477–1516 m, with similar burial depths and a crater thickness of ∼2.1 m, and are covered by 4.0 m of overlaying sediments (Fig. 2f and g). However, at 1496 m, the Zhurong rover took a U-turn (Fig. 1a); thus, the same crater was imaged twice. The image at the U-turn point exhibits a perfectly symmetrical pattern, confirming that the imaging results are correct (Fig. 2f).

### Typical structure and features revealed at decimeter and centimeter scales

**Figure 3.**
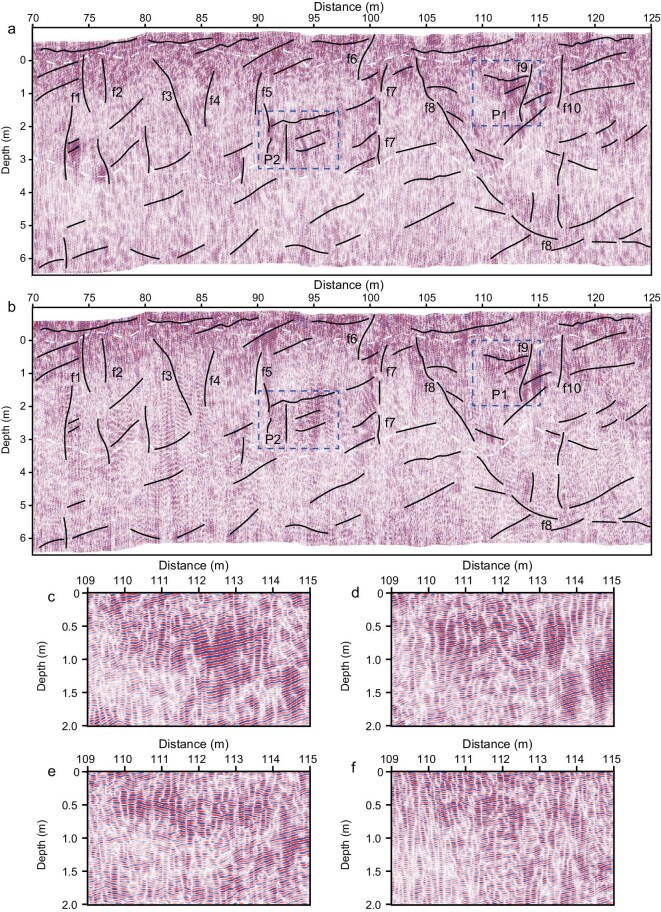
Multicomponent polarized image. Data from Sols 26–30 (profile 4 in Fig. 2a) indicates the quad-polarized image with AGC formed by HV, HH, VH, and VV at the decimeter scale. (a, b) Represent HV and HH images. The slopes with inclinations ranging from 8^o^ to 15^o^, highlighted by black lines, are the dominant features in the middle and bottom layers. Additionally, the slopes labeled f1 to f7, along with f9 and f10, indicate fractures primarily distributed within the middle layer. The concave-up fault tending northward, labeled f8, extends from the top of the middle layer to the bottom layer and gradually disappears as it reaches the bottom layer. The two dashed boxes in panel (a) indicate areas interpreted as platy rocks and duricrusts found on the surface. As an example, Fig. 3c–f depicts the HV, HH, VV, and VH centimeter-scale quad-polarized images, respectively, of the P1 region. The two dashed white lines indicate the layer boundaries, as shown in Fig. 2.

**Figure 4.**
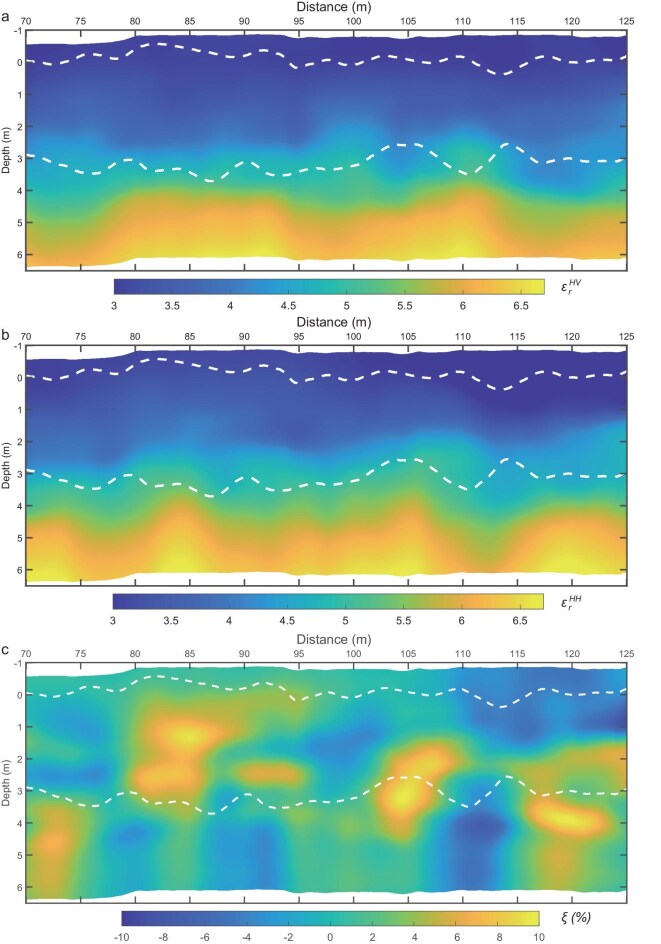
Dielectric permittivities of HV and HH modes. The dielectric permittivity values for the top, middle, and bottom layers, separated and delineated by white dashed lines, range from 3.0–3.5, 3.5–5.0, and 5.0–6.5 for the HV and HH data corresponding to (a) and (b), respectively. The dielectric constants of HV and HH are stratified into three layers, with some local variations. The white dashed lines indicate the boundary layers, which are also represented by the two black lines in Fig. 2a, c), and illustrates the anisotropy of dielectric permittivity, capturing variations in layer-sediment orientation through HH and HV measurements.

Radar data imaging is highly sensitive to the orientation of subsurface interfaces. Multiple-channel radar antennas can compensate for imaging gaps caused by a single channel [30]. The average trace amplitude (ATA) provides an estimate of the maximum penetration time or depth of the polarized wave (Fig. 4S; Method). The signal of the HV channel is stronger than that of the HH channel, demonstrating that the subsurface structure of the Zhurong landing site is complex. Polarization changes can occur in irregular rocks, inclined planes, and anisotropic media. Quad-polarized radar images acquired between Sols 26 and 30 (corresponding to profile 4 in Fig. 2a), including HV and HH polarizations (Fig. 3a, b) to a maximum depth of 7 m, and VH and VV polarizations (Fig. S5a and b) to a depth of 5 m, resolve the near-surface stratigraphy and enable high-resolution characterization of the subsurface structure in the vicinity of the landing site. The characteristics of this segment are typical structures that effectively represent the features of a 1.9 km traverse. The top layer (Fig. 3a and b) in the HV and HH images displays a local horizontal pattern with relatively weak scattered signals (Fig. 2a, without AGC). This layer is speculated to be an unconsolidated loose sediment layer formed by weathering and meteorite impacts into sedimentary rocks. The layer is mixed with a small amount of wind-blown sand and rock fragments with centimeter-sized pebbles and preserves the original sedimentary layering, which is supported by surface observations of the Zhurong rover and a hole excavated by the Zhurong retro rocket [26]. The HV image differs slightly from the HH image, even though both share the same horizontally polarized transmitting antenna. The difference arises from the vertical polarization of the receiving antenna in the HV and HH modes and can be used to detect thin, inclined stacked layers and fracture orientations.

The widespread presence of slopes in HV and HH images with inclinations ranging from 8^o^ to 15^o^, extending from the middle to the bottom layers and marked by black lines (not labeled with numbers) in the polarized radar image, indicates northward-oriented reflectors (Fig. 3). These slopes are the dominant features in the middle and bottom layers. Additionally, the features labeled f1 to f7, as well as f9 and f10, represent fractures primarily distributed within the middle layer. A southward-dipping, concave-up fault labeled f8 extends from the top of the middle layer to the bottom layer, gradually flattening out as it reaches the bottom layer. The fractures and the concave-up fault were possibly caused by a meteorite impact, which reworked the middle layer. Low-frequency data [21] from 10 to 13 m exhibit dielectric constants and a weak reflectivity approximately the same as those of the bottom layer imaged by high-frequency data. Although there is a gap between 7 and 10 m in depth, where neither low- nor high-frequency data provide effective resolution, reflectivity and dielectric permittivity data suggest that the bottom layer imaged by high-frequency data and the 10–13 m layer identified by low-frequency data likely belong to the same formation. This indicates that the bottom layer extends to depths of from 7 m to 13 m.

The high-frequency radar data resolves subsurface features down to a depth of 7 m. The dielectric permittivity values for the top, middle, and bottom layers range from 3.0–3.5, 3.5–5.0, and 5.0–6.5, respectively, based on the HV and HH data (Fig. 4, Fig. S6, Methods). Although scattered rocks and irregularly inclined interfaces can alert to the polarization direction of the wave, fractures are the dominant source of anisotropy in the scattered wavefield. The anisotropy of dielectric permittivity, derived from HH and HV measurements, can detect anisotropies to the inclination of layered rocks and approximately vertical fractures, which leads to azimuthal anisotropy [31,32] (Fig. 4c). This property is critical for analyzing fractured structures, as it reflects variations in dielectric permittivity and polarization. The top layer, between 70 and 110 m horizontally, shows no significant anisotropy because its horizontal layering cannot be resolved using HH and HV polarizations. In contrast, a slight anisotropic response is observed between 110 and 125 m, where the layers dip gently. The middle layer displays stronger anisotropic characteristics, with features that suggest a few northward inclinations of layer rocks and fractures, consistent with the imaging profile. In contrast, the bottom layer exhibits weaker anisotropy, ranging from −5% to 5%, except for two localized regions reaching ∼15%. The interval between 3.1 to 4.5 m depth and 116 to 124 m horizontally coincides with concave-up fault structures in the bottom layer. These zones likely represent highly anisotropic media, possibly indicating the presence of plastic features.

## DISCUSSION

### Possible aqueous activity during the middle-late Amazonian

The thin-layering features identified in the high-frequency profiles suggest there is a sedimentary environment. Wind-blown sand can create sand sheets [33,34]. However, unlike the findings made by the Opportunity rover at Meridiani Planum [35], where extensive sand sheet deposits were observed on the surface, Zhurong cameras did not detect similar wind-formed layered sediments at its landing site. Instead, NaTeCam identified bedding laminations, which are commonly found near coastlines and beaches [18]. Additionally, high- and low-frequency radar images show no signs of dunes under the subsurface, making an aeolian (wind-driven) origin for these deposits less likely. Low-frequency radar data indicate an absence of lava flows in the subsurface of the Zhurong landing site down to at least 80 m [21]. The Zhurong landing site is distant from the two nearest volcanic vents, namely, Elysium Mons (25.02°N, 147.21°E) and Syrtis Major Planum (8.4°N, 69.5°E), which are ∼1760 km and 2086 km away, respectively. This makes it impossible for lava to have flowed to the Zhurong landing site area. Observations of surficial rock blocks by MarSCoDe and NaTeCam did not identify metamorphic rocks [16]. This suggests that metamorphic formations are unlikely at the Zhurong rover landing site, as their formation requires high temperatures and pressures. However, the presence of pyroclastic material, such as volcanic ash, and the possibility of metamorphic debris formation due to meteorite impacts cannot be ruled out entirely.

The most likely materials in the landing area are sedimentary rocks. The loss tangents at the Zhurong landing site are 0.0076 for HV and 0.0078 for HH (Fig. S7, Methods), both of which are lower than the 0.014 measured by the Perseverance rover in Jezero Crater [36]. This suggests that the lava-dominated region at the Perseverance landing site has a higher attenuation than the near-surface sedimentary strata at the Zhurong landing site. More than 278 mud volcanoes have been found near the Zhurong landing site [37], with the closest one ∼6.6 km away. Their widespread distribution suggests that the area has experienced past water activity. Low-frequency radar data reveal that two resurfaced sediment layers at the landing site were subjected to flooding: one at a depth of 10 to 30 m, dating to ∼1.6 billion years ago, and another at 30 to 80 m, dating to 3.2 billion years ago [21].

The MarSCoDe instrument on the Zhurong rover detected hydrated materials, including gypsum, bassanite, and epsomite, on finely layered sediment slabs [16] and bedding laminations [18] on the surface (Fig. 1b). Our high-frequency images also revealed centimeter-scale, buried thin-layered sediment interpreted as platy structures or duricrusts within the middle layer, suggesting they formed in a water-based sedimentary environment (Fig. 3). The concave-up faults extending from the top to bottom layers tend to become horizontal at the base, exhibiting characteristics of sediment plastic deformation [38], which likely indicates salt-rich or clay-rich deposits in the bottom layer. A possible geological scenario involves a sequence of evaporitic sediments, where salt-rich layers (e.g. halite or gypsum) are interbedded with silty or clayey deposits. The relatively low dielectric permittivity of salts (∼5–6) compared with basaltic regolith or clay-bearing sediments (∼7) enhances dielectric contrast, which can increase anisotropic effects and produce measurable differences between co- and cross-polarized radar channels. Although MarSCoDe did not detect clay rocks on the surface [16,17], the inverted dielectric constant in the bottom layer is ∼6.5, consistent with the permittivity of salt-rich and clay-rich mineral deposits in this layer [38,39]. The anisotropic parameter, ranging from −5% to 5% in the bottom layer, exhibits weak anisotropy, indicating a salt-rich deposit, and suggesting past aqueous activity.

Two buried craters identified in the middle and lower layers (Fig. 2) provide evidence of distinct sedimentary periods. The lower crater is ∼2.1 m thick and is overlain by 4 m of subsequent deposits from the crater edge to the surface. Not only are 4-m-thick overlain sedimentary materials filling up the crater, but the large area of the Zhurong landing site is also covered. Given the shallow burial depth, the sedimentary characteristics, including the presence of gypsum-bearing rocks, bedding laminations observed both in outcrops and at the surface [16,18], and centimeter-scale layering detected in high-frequency radar imagery (Fig. 3), strongly suggest a water-deposited environment [40,41] likely formed under a shallow body of water. The thickness of the overlying materials and the dimensions of the craters further indicate that the Zhurong landing site was once part of a shallow aquatic environment during its formative period [42,43]. This interpretation is supported by the well-preserved morphology of the two buried craters.

A key feature observed in high-frequency images is the widespread presence of parallel-inclined, northward-trending slopes at angles of 8° to 15° over a distance of 1.9 km (Fig. 3a) in the middle and bottom layers. These inclinations align with identified slopes ranging from 6° to 20° at depths of 10 to 30 m, which have been interpreted as a prograding shoreline based on low-frequency data [24]. This further supports the hypothesis that the Zhurong landing site was located in a shallow water environment during crater formation. During high-obliquity periods, ice from the polar ice cap is redistributed toward the equator, and during lower-obliquity periods, it sublimates back into the atmosphere and returns to the north pole [44]. This cycle generates aqueous activity that plays a critical role in controlling the sediment process during the middle to late Amazonia [44–46]. Although no traces of a local lake or ocean have been observed at the surface of the Zhurong landing site, cyclical processes may have caused ground ice to melt and groundwater to rise, potentially leading to temporary flooding during periods of high obliquity in the middle to late Amazonian periods.

**Figure 5.**
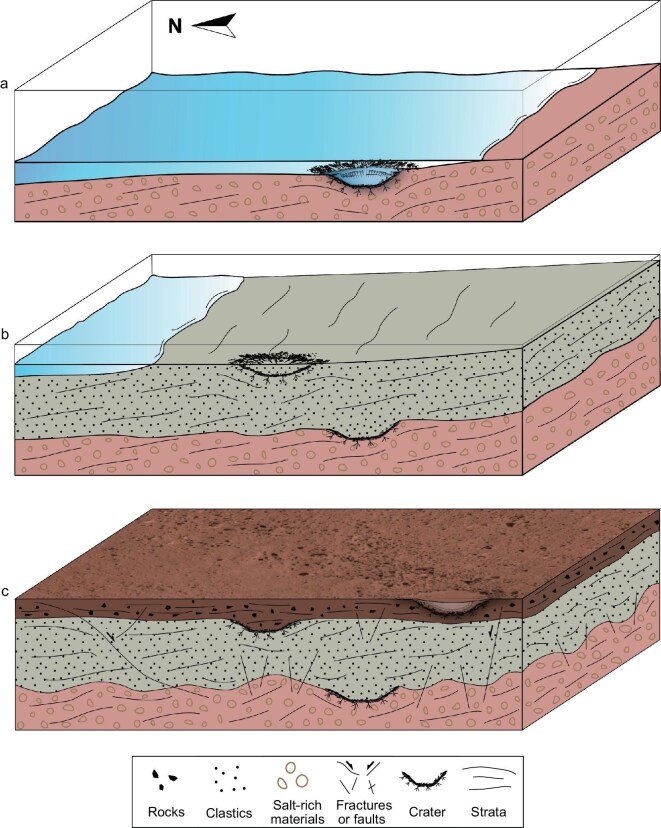
Evolution of near-surface sedimentary formations at the Zhurong landing site. The three layers exhibit weathering, strong reflectivity, and salt-rich or clay-rich materials. Slopes with inclinations ranging from 8^o^ to 15^o^ are widespread, extending from the middle to the bottom layers and indicating northward-oriented reflectors. (a) In Stage 1, the bottom layer formed in an aqueous activity environment during the middle to late Amazonian period. (b) In Stage 2, the middle layer developed through the deposition of sediments from local lakes or groundwater rise, clastic deposits, the formation of slopes and fractures, and craters. (c) In Stage 3 the top layer represents a weathered sedimentary unit characterized by hydrated rocks. This layer was likely formed through the weathering of the middle layer, erosion, and clastic sediment deposition.

High-frequency radar data provide higher-resolution imaging than previous Martian radar studies, revealing buried craters, extensive parallel slopes, fractures, faults, and subsurface platy structures. The geological features identified by high-frequency data reveal not only scattered objects but also a variety of complex geological structures in the immediate subsurface. The widespread slope structures, <10 m in depth, identified in high-frequency data in the middle and bottom layers align well with ancient shorelines at depths of 10 to 30 m identified in low-frequency data. These high-resolution observations provide additional support for the hypothesis that the Zhurong landing site was initially formed in a shallow aquatic environment. Crater-counting analyses constrain the age of the subsurface materials, indicating that the bottom layer formed ∼757 million years ago. In addition, low-frequency radar data reveal evidence of an earlier flooding event at a depth of 30 m, dated to ∼1.6 billion years ago. Our findings suggest that a resurfacing event likely occurred during the middle to late Amazonian period. Furthermore, aqueous activity persisted at the Zhurong landing site as recently as this period (Fig. 5).

While we have demonstrated that the Zhurong rover landing site is situated in a sedimentary geological setting and present that aqueous activity likely occurred in the immediate subsurface of the region, the age of this activity remains uncertain, particularly near the surface, and requires further verification. Determining the timing of this aqueous activity is crucial to our conclusions, and we anticipate that more reliable age-dating methods, such as sample return missions, will be necessary.

## METHODS

### RoPeR high-frequency-multipolarization data processing

The high-frequency channel of the Zhurong rover has a frequency bandwidth ranging from 450–2150 MHz, which is used to survey the immediate Martian subsurface, with a resolution of a few centimeters and a depth range of several meters. The transmitting and receiving antennas on the rover are spaced 420 mm apart, with heights of 345 mm and 306 mm, respectively [2]. With different polarimetric antennas, the high-frequency channel has four modes: HH, HV, VH, and VV. The transmitting interval is 0.05 m, and the data sampling rate is 0.1147 ns. The HV (Fig. S2a), HH, VV, and VH components of the data from the high-frequency channel were processed via time zero correction (18 ns), background removal, multiple reflections removal, bandpass filtering, random noise attenuation, topographic correction, diffraction velocity analysis, dielectric permittivity estimation, automatic layer boundary identification, loss tangent estimation, and Kirchhoff migration (Fig. S2). The data processing consisted of the following steps.

1. Background subtraction. Transmitter reverberation, electromagnetic interference, a time-synchronous system, and surface reflections can generate background artifacts in data. Average trace removal can be performed by calculating the mean of a selected window of traces, and then subtracting this average from the raw data, thereby reducing horizontal banding in the dataset (Fig. S2b).
2. Multiple reflections removal (Ring noise). Ground-penetrating radar (GPR) waves transmitted into the subsurface can generate multiple reflections, particularly near the surface. These are commonly referred to as ringing noise in GPR data. Ringing noise typically appears as horizontal, quasi-periodic, and coherent energy across multiple traces, often resulting from antenna ringing, system response, or direct-coupled surface waves. To attenuate these ring-related multiples, predictive deconvolution and surface-related multiple suppression techniques are applied [47] (Fig. S2b).
3. Bandpass filtering. The quad polarimetric data are filtered to isolate the data bandwidth from 450–2250 MHz by bandpass filtering, thereby removing high- and low-frequency noise. We used a method that provides general-purpose zero-phase filtering and uses sine-squared tapering between frequency values of different amplitudes [48] (Fig. S2c).
4. Random noise attenuation. Random noise was suppressed to enhance the visibility of the whole profile. We used the FX deconvolution method to remove random noise [49] (Fig. S2d).
5. Kirchhoff time migration. The purpose of migration is to remove distortions, dip displacements, and out-of-line reflections. After migration, the diffractions are collapsed to reveal a more realistic subsurface structure. Migrated profiles were obtained via Kirchhoff time migration (Fig. S2e) and then transformed to the depth domain (Fig. S2f) on the basis of the dielectric permittivity estimated via diffraction wave velocity analysis [50].
6. Topographic correction. The acquired radar data do not reflect topographic elevation changes along the Zhurong rover track, and these changes can be corrected by moving migrated traces upward and downward on the basis of the relative elevation data (Fig. S2f).
7. Automatic gain control (AGC). As the propagation depth increases, the energy of the radar echoes decreases gradually. AGC is a gain recovery method in electromagnetic processing and was applied to the data on a trace-by-trace basis via a sliding time or depth window to increase the energy of radar echoes from deep reflectors [48] (Fig. S2g).

### Layers estimated via statistical zonation

The image in Fig. 2a displays a discernible pattern of amplitude variation characterized by a sequence of weak–strong–weak transitions extending from the surface to depth. Notably, the imaging profile exhibits a distinct zonal distribution, indicative of varying physical properties. This observation led us to use an automated methodology to discern the demarcated boundaries between these zones [51]. To achieve this, the electromagnetic wave data-driven imaging outcomes were subjected to statistical analysis by employing variance procedures. The aim of our approach was to establish optimal subdivisions for these zones, with the goal of minimizing within-zone variance while maximizing between-zone variance. These results are represented in histograms. Using a Gaussian distribution function, we fit the histograms and calculated the average depth of the first and the second boundaries (Fig. S3).

### Power of the average trace amplitude

The process of generating an average trace amplitude (ATA) plot involves plotting the average response characteristics of all traces over time to measure the ring noise level [52]. To create the ATA plot, all traces from a ground-penetrating radar (GPR) line are rectified, converting negative signal amplitudes into positive amplitudes, which allows clearer representation of the signals. The averaged rectified data incorporate both noise and signals, resulting in an informative presentation. The ATA plot is valuable for evaluating and quantifying the noise and penetration depth of GPR signals. The depth at which the GPR signals reach the same amplitude as the background noise is defined as the GPR depth of penetration. This depth measurement is essential for assessing the capabilities of the GPR system and understanding how deeply radar waves can penetrate the subsurface to detect buried objects or structures (Fig. S4).

### Dielectric permittivity estimated via diffraction

The dielectric permittivity, which is directly related to rock properties, is a parameter representing how much a medium slows an electromagnetic wave compared with the speed of light. The following equation gives the radar propagation velocity in a medium:


(1) \begin{eqnarray*}
v = \frac{c}{{{{\sqrt \varepsilon }}_r}},
\end{eqnarray*}

where *c* is the propagation velocity of electromagnetic waves in free space (3 × 10^8^ m/s) and ${\varepsilon }_r$ is the dielectric permittivity relative to free space. Diffraction velocity analysis [53] was conducted to estimate the dielectric permittivity for the HV and HH datasets. The diffracted velocity potential was found for groups of electromagnetic waves gathered by scanning a hyperbolic region (Fig. S6), resulting in M wavelet time samples and N horizontal spacing intervals. The output from one set of velocity analyses was a table of numbers as a function of velocity versus two-way zero-offset time (velocity spectrum). These numbers represented a measure of signal coherency and semblance along the hyperbolic trajectories governed by velocity, offset, and travel time.

Diffraction velocity analysis (Fig. S6) was used to obtain a velocity model for the HV and HH data. The velocity spectra corresponding to the dielectric permittivity were derived from common diffracted electromagnetic wave gatherings associated with hyperbolic curves. The horizontal axis in each spectrum represents the scan velocity, with a range of 0.05–0.30 m/ns, and the vertical axis represents the two-way zero-offset time from 0–80 ns. Red indicates the maximum coherency measure. The curve in each spectrum represents the velocity function based on the selected maximum coherency values associated with diffracted waves. The scanning region comprised 35 diffracted wave traces in the horizontal plane and 5 vertical samples determined by the dominant frequency for the Zhurong rover data. The velocity panel was transformed into the internal velocity model and then into the dielectric permittivity model via equation (1).

### Electromagnetic anisotropy

An electromagnetic H polarization splits into fast and slow velocity waves when H propagates through stacked inclined slabs and approximately vertical fractures, leading to azimuthal anisotropy in electromagnetic media. Under the common assumption of isotropic media and horizontal interfaces at normal incidence, co-polarized channels (HH and VV) are expected to provide clearer stratigraphic signals than cross-polarized channels (HV and VH). While oblique planes, scattered rocks, and irregular interfaces can scatter energy in multiple directions, thereby generating cross-polarized components. The imaged structure of the Zhurong landing site subsurface reveals approximately vertical fractures and stacked slab features, which create waves acquired by the H and V receiving antennas when a polarized wave propagates through this anisotropic medium. The structural medium is characterized by azimuthal anisotropy. The azimuth reflection coefficient and density are related to the properties of the filling materials and fluid. Because the H polarization of the transmitting antenna is parallel to the surface and the receiving antenna records the horizontal component of the scattered field, the rover radar is particularly sensitive to media exhibiting horizontal transverse isotropy (HTI). This configuration enhances the radar’s response to azimuthal variations in electromagnetic properties, making it well-suited for detecting fracture-induced anisotropy or preferentially aligned inclined subsurface features.

Water ice, layered strata, parallel fractures, and subsurface hydrated minerals all tend to have anisotropic characteristics. In contrast, when a medium has a stacked pattern defined by scattered rocks or rock blocks, the scattering waves exhibit no directionality, and the imaged patterns are almost identical for different polarizations. Dielectric permittivity can be used to estimate the anisotropy coefficient and the azimuthal dielectric constant via equations (2) and (3) [54,55]:


(2) \begin{eqnarray*}
\xi (\% )\,{\mathrm{ = }}\,\frac{{\sqrt {{\varepsilon }_{HH}} - \sqrt {{\varepsilon }_{HV}} }}{{{{\bar{\varepsilon }}}_i}}*100,
\end{eqnarray*}

(3) \begin{eqnarray*}
\bar{\varepsilon } = (\sqrt {{\varepsilon }_{HH}} + \sqrt {{\varepsilon }_{HV}} )/2.
\end{eqnarray*}

In our data, the H and V polarizations did not precisely correspond to fast and slow polarizations in the media because of the presence of partly vertical polarization recordings for high-frequency data. Nonetheless, they still reliably measured the difference in dielectric permittivity in H and V polarizations resulting from the structural directionality (Fig. 4c). In our study, the radar operates at frequencies up to 2.15 GHz, corresponding to a wavelength of ∼8 cm in the Martian regolith (assuming a relative permittivity of ∼4 at the near surface). The dominant frequency is ∼1 GHz, corresponding to a wavelength of ∼16 cm. This implies that, for the medium to behave as an azimuthally anisotropic continuum, the fracture spacing would need to be smaller than this wavelength.

### Loss tangent estimated via the centroid frequency-shift method

Loss tangent is a parameter used to measure the attenuation of electromagnetic waves through a medium [36]. When a medium is lossy, the centroid frequency of the radar signal experiences a downshift during propagation. On the basis of this phenomenon, the loss tangent of the lossy medium can be estimated by measuring the descent rate of the echo signal’s centroid frequency [56]:


(4) \begin{eqnarray*}
\tan \,\delta = - \displaystyle\frac{1}{{\sigma _t^2\pi }}\frac{{\Delta {f}_c}}{{\Delta t}},
\end{eqnarray*}

$\frac{{\Delta {f}_c}}{{\Delta t}}$ represents the slope of the variation in the centroid frequency with propagation time; this slope can be determined via least-squares linear regression; $\sigma _t^2$ is the variance of the transmitted spectrum. The instantaneous centroid frequency *f_c_* can be calculated via the following equation:


(5) \begin{eqnarray*}
{f}_c(t) = \frac{{\int{{f \cdot F(f,t)df}}}}{{\int{{F(f,t)df}}}},
\end{eqnarray*}

where $F(f,t)$ is the time-frequency spectrum obtained via short‒time Fourier transform (STFT). The RoPeR high-frequency signals are divided into segments with a spacing of 10 m. For each segment, we applied the centroid frequency-shift method to estimate an independent loss tangent. Finally, we fit the distribution of the estimated loss tangents to a Gaussian curve and obtained the average loss tangent (Fig. S7).

## DATA AND MATERIALS AVAILABILITY

The data reported in this work are available from ScienceDB System. The Mars Rover Penetrating Radar (RoPeR) data used in this study are available from the website (https://www.scidb.cn/s/uuA3M3). Data download problems and other datasets generated and analyzed in this study are available from the corresponding author upon request.

## Supplementary Material

nwaf505_Supplemental_File
