# Supplementary material for: Multipolarized radar reveals shallow subsurface structure and middle-late Amazonian aqueous activity in Utopia Planitia, Mars
Source: Natl Sci Rev. 2025 Nov 14;12(12):nwaf505. doi: 10.1093/nsr/nwaf505 (PMC12696870; doi:10.1093/nsr/nwaf505)
Supplement: nwaf505_Supplemental_File [file nwaf505_supplemental_file.pdf]

Multipolarized radar reveals shallow subsurface structure and middle-late Amazonian aqueous activity in Utopia Planitia, Mars

Yike Liu[1]*†, Tianfan Yan[1]†, Xiaoguang Qin[2]†, Ling Chen[2], Jinhai Zhang[3], Yang Liu[4], Yangting Lin[3], Fuyuan Wu[2], Ross N. Mitchell[2,3], Zhendong Zhang[1], Jiangjie Zhang[1], Haiwei Wang[1], Chao Li[3], Yibo Wang[1], Bin He[1], Yikang Zheng[1], Lei Zhang[3], Juan Li[3], Kaichang Di[5], Wenhui Wan[5], Honglei Lin[3], Jiangang Wang[3], Jinlai Hao[2], Xin Wang[2], Pan Zhao[3], Xu Wang[2], Yongxin Pan[3,6]

[1]Key Laboratory of Deep Petroleum Intelligent Exploration and Development, Institute of Geology and Geophysics, Chinese Academy of Sciences; Beijing 100029, China.

[2]State Key Laboratory of Lithospheric and Environmental Coevolution, Institute of Geology and Geophysics, Chinese Academy of Sciences; Beijing 100029, China.

[3]Key Laboratory of Earth and Planetary Physics, Institute of Geology and Geophysics, Chinese Academy of Sciences; Beijing 100029, China.

[4]State Key Laboratory of Space Weather, National Space Science Center, Chinese Academy of Sciences; Beijing 100190, China.

[5]State Key Laboratory of Remote Sensing Science, Aerospace Information Research Institute, Chinese Academy of Sciences; Beijing 100101, China.

[6]College of Earth and Planetary Sciences, University of Chinese Academy of Sciences, Chinese Academy of Sciences, Beijing 100049, China

†These authors contributed equally to this work: Yike Liu, Tianfan Yan, Xiaoguang Qin

*Corresponding author. Email: ykliu@mail.iggcas.ac.cn

This file includes:

Figs. Supplementary Figure 1 to Figure 7

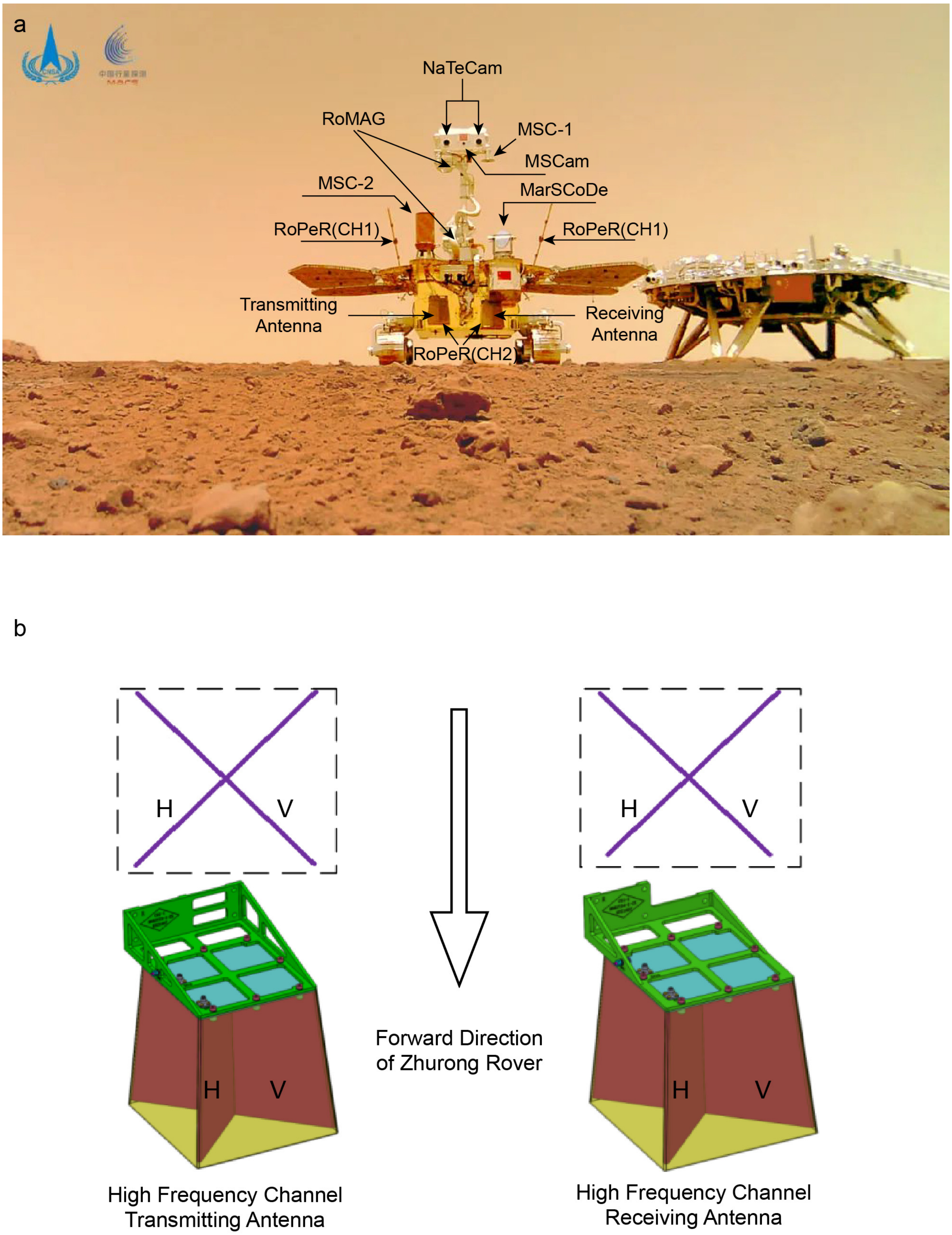
a
NaTeCam
RoMAG
MSC-1
MSCam
MSC-2
MarSCoDe
RoPeR(CH1)
RoPeR(CH1)
Transmitting Antenna
Receiving Antenna
RoPeR(CH2)
b
H
V
H
V
Forward Direction of Zhurong Rover
H
V
H
V
High Frequency Channel Transmitting Antenna
High Frequency Channel Receiving Antenna

Figure S1. Scientific payloads mounted on the Zhurong rover. a Picture of the rover (left) and the lander (right) taken by the WiFi camera released by the lander. Credit: China National Space Administration (CNSA). NaTeCam: Navigation and Terrain camera. RoMAG: Mars Rover Magnetometer. MSCam: Multispectral Camera. MSC-1: Mars Climate Station (Wind field and sound probe). MSC-2: Mars Climate Station (Air temperature and pressure probe). MarSCoDe: Mars Surface Component Detector. RoPeR-Ch1: Mars Rover Penetrating Radar (channel 1). RoPeR-Ch2: Mars Rover Penetrating Radar (channel 2). **b**, RoPeR-Ch2 (Ch2) is a high-frequency quad-polarization antenna (HH, HV, VV, VH) with a frequency range from 450–2,150 MHz.

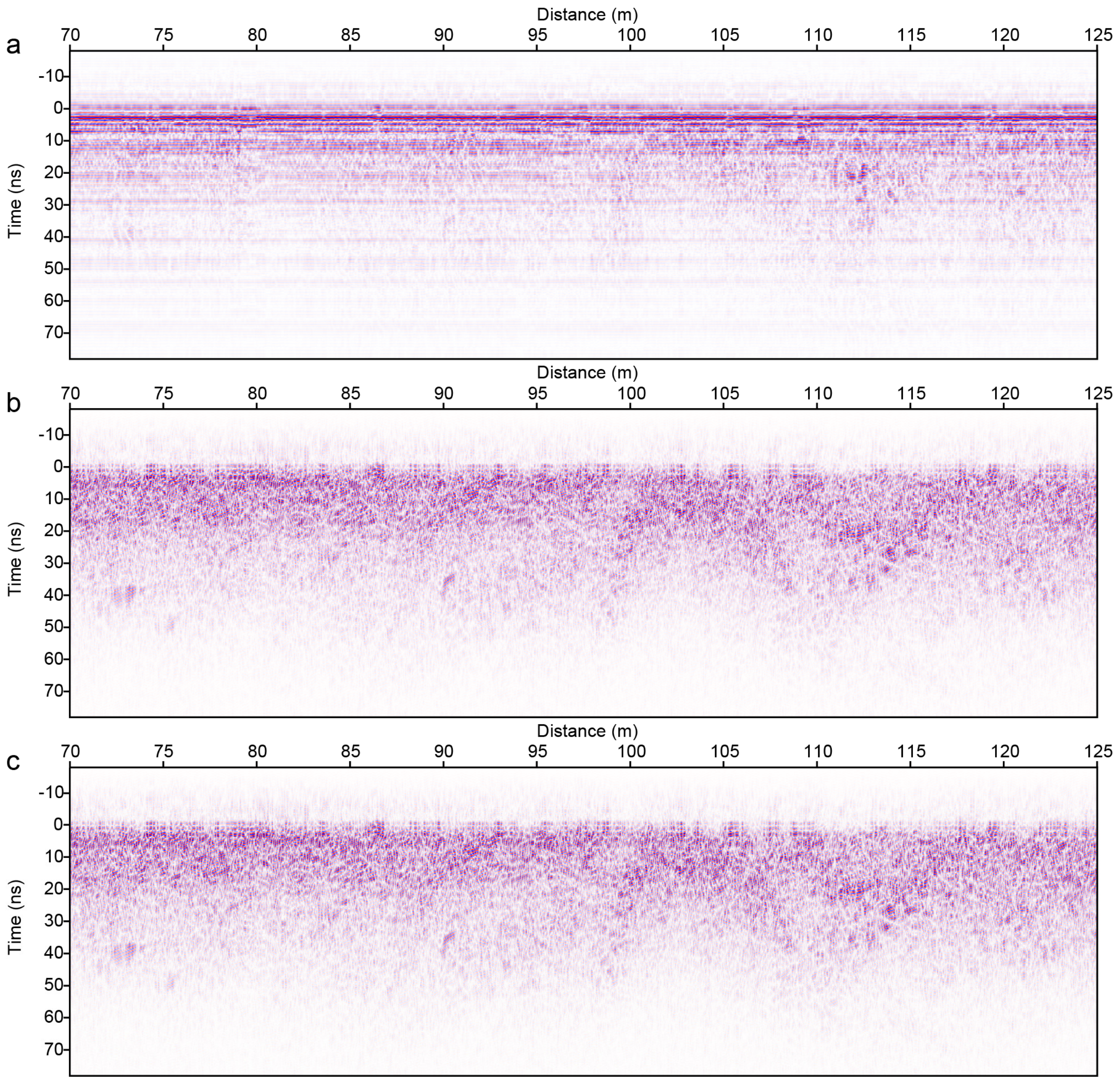
a
b
c
Distance (m)
70
75
80
85
90
95
100
105
110
115
120
125
Time (ns)
-10
0
10
20
30
40
50
60
70

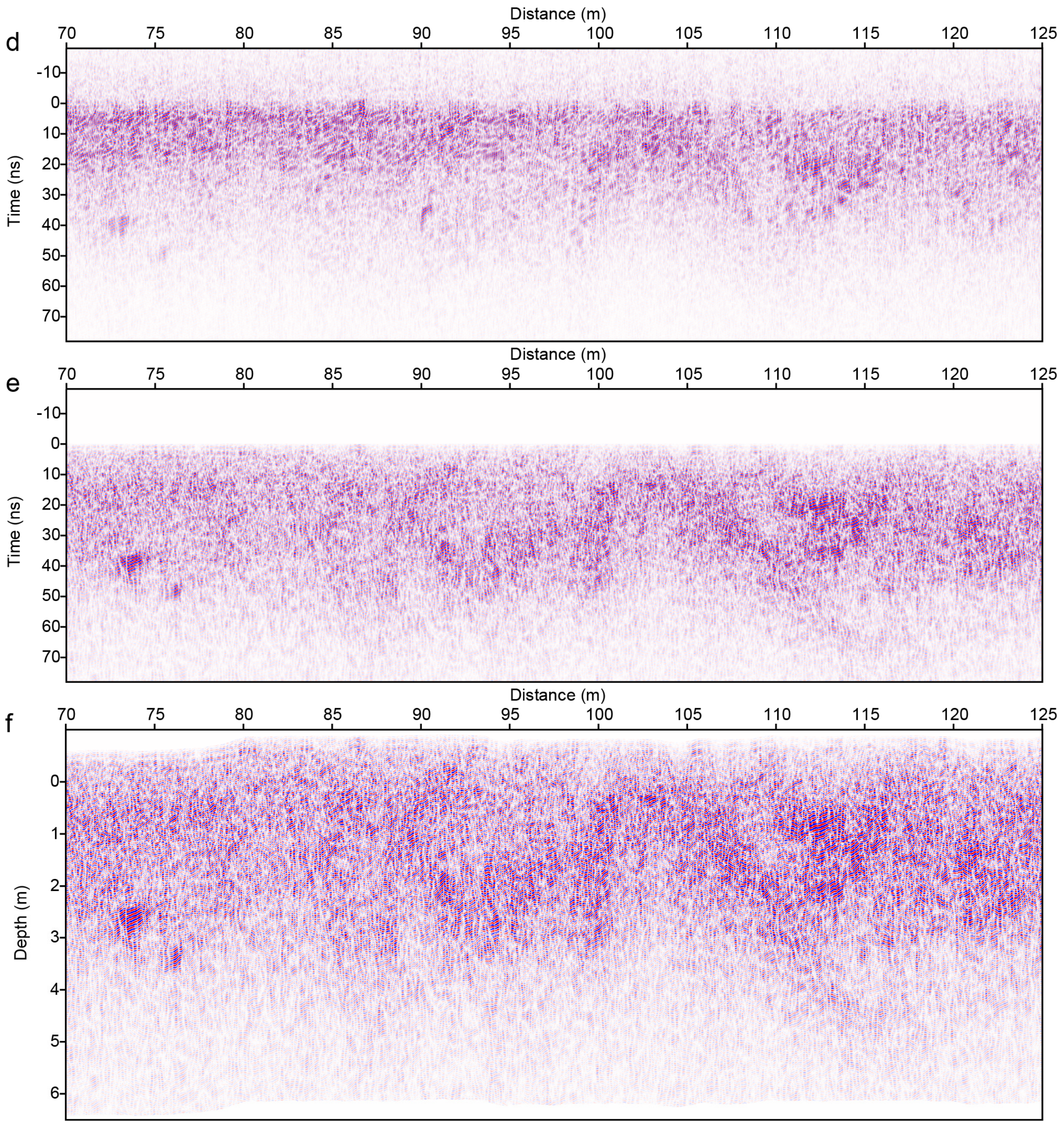
d
Distance (m)
70
75
80
85
90
95
100
105
110
115
120
125
Time (ns)
-10
0
10
20
30
40
50
60
70
e
Distance (m)
Time (ns)
f
Distance (m)
Depth (m)
0
1
2
3
4
5
6

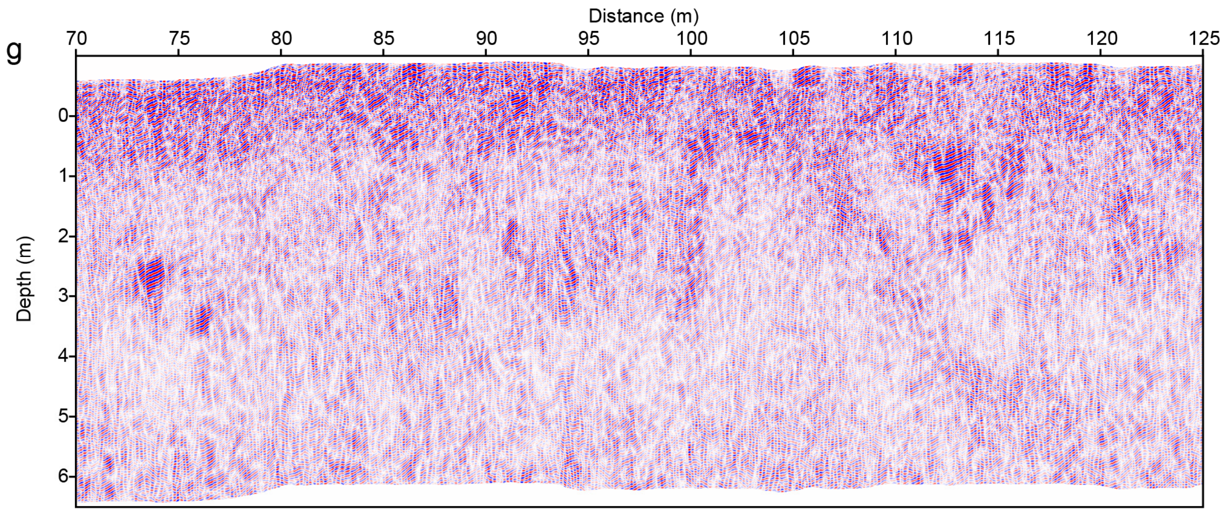

Figure S2. Illustration of the sequence of data processing steps. a, HV raw data. **b**, Background removal. **c**, Bandpass filtering. **d**, Random noise removal. **e**, Kirchhoff time migration. **f**, Depth migration converted from the time domain and elevation correction. **g**, Final image with AGC and random noise attenuation.

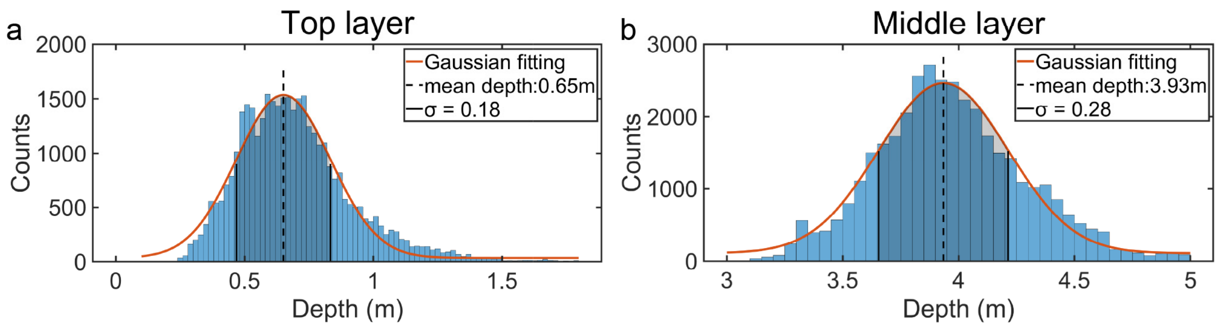

Figure S3. Histograms of the depths of the top and middle layers. a, Thickness (depth) changes in the top layer from 0.47–0.83 m (shadowed area), with an average of 0.65 m. **b**, Depth changes in the middle layer from 3.65–4.21 m (shadowed area), with an average of 3.93 m. The σ represents the standard deviation that is the estimate of the depth ranges for the layers.

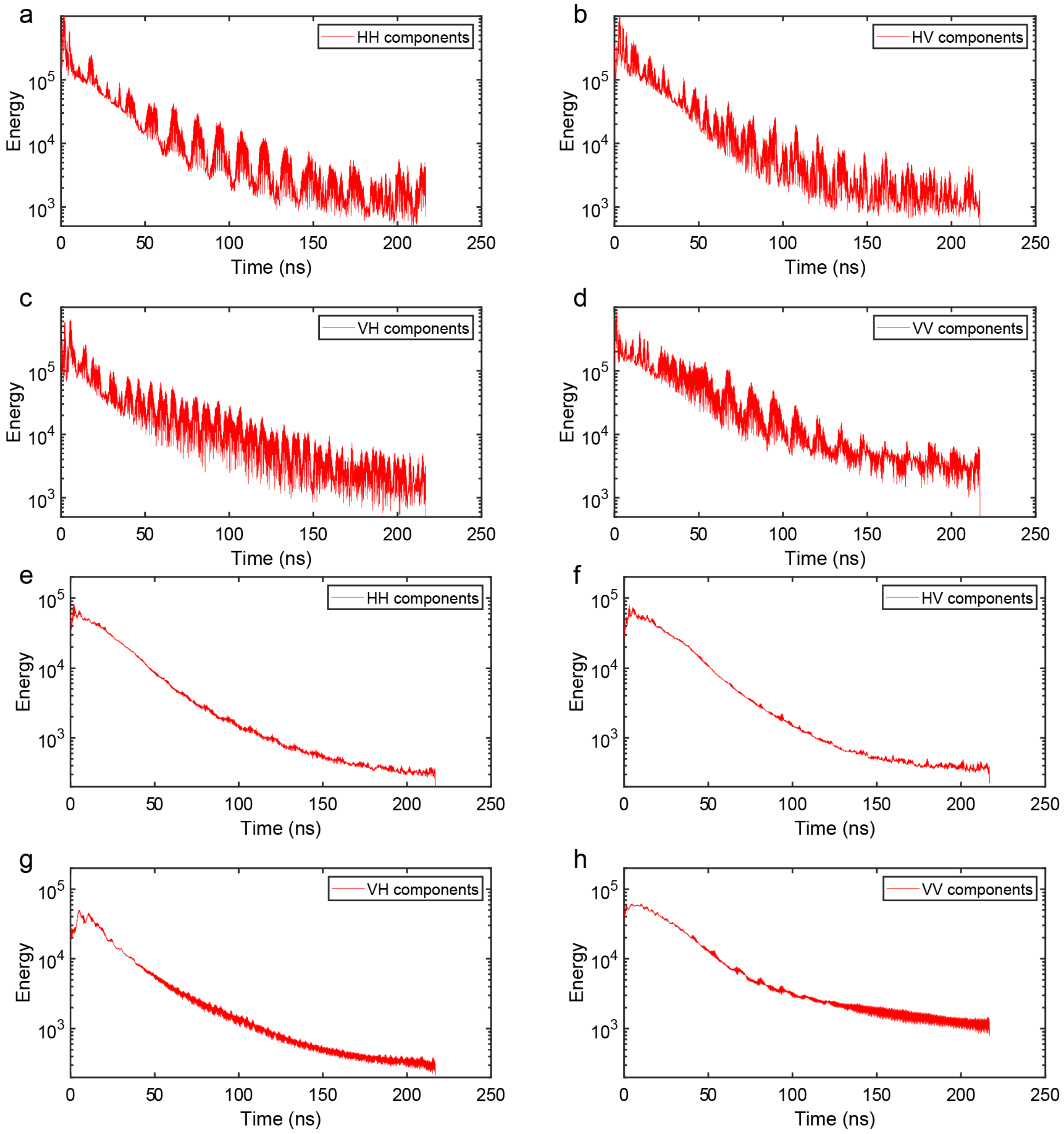

Figure S4. Average trace amplitude (ATA) plot of the HH, HV, VH, and VV data before and after noise removal. In the quad-polarized data, the dominant noise is represented by a ring pattern, exhibiting distinct characteristics in different polarizations in the ATA plots. Panels **a, b, c,** and **d** show the ATA plots before noise removal. Copolarization HH (**a**) and VV (**d**) data show evident

discrete wave packets associated with the ring noise, whereas cross-polarized data HV (**b**) and VH (**c**) display high-frequency interval ring patterns compared with the copolarized HH and VV data. These ring noise characteristics raise concerns about their origin, as they might be attributed to the rover or equipment rather than the subsurface. The background levels are achieved at approximately 170 ns (14.03 m) for quad-polarization data when the noise level curve becomes flat, indicating a stable baseline, particularly for HH and HV data that display background time positions. Panels **e, f, g,** and **h** are ATA plots after the noise is attenuated for quad-polarization data. The minimum signal noise level appears at approximately 100 ns (8.25 m), corresponding to the penetration time or depth where valid signals can be reliably detected before this point for HH (panel **e**). Panel **f** is the background level, which is at 170 ns (14.03 m), demonstrating good noise removal for the HV data. In Panel **g**, VH has heavy residual noise, and the valid signal reaches only 61 ns (5 m). In Panel **h**, VV has better noise removal than VH does, and valid signals can last up to 70 ns (5.78 m). These observations are crucial for signal interpretation and noise assessment, assisting in accurately analyzing and understanding subsurface conditions.

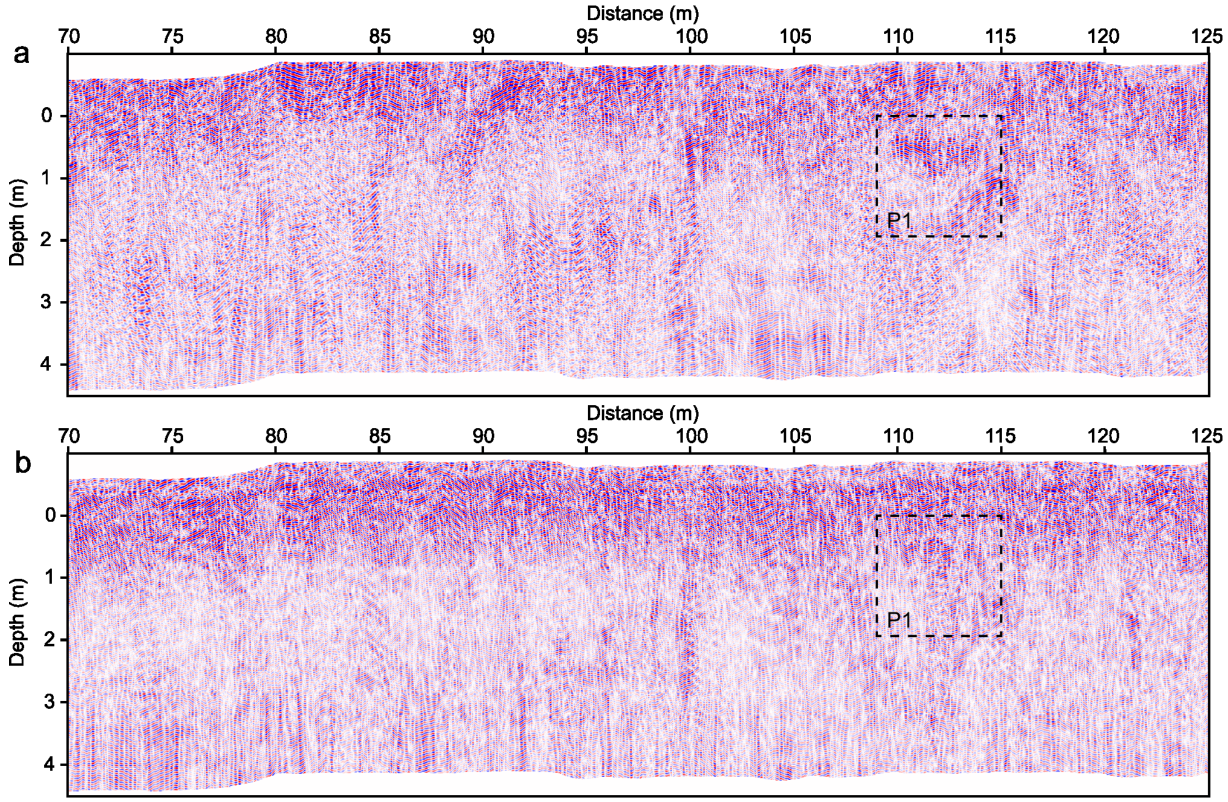

Figure S5. Multiple-component polarized images taken during Sols 25 to 30 with AGC. Panels **a** and **b** display VV- and VH-polarized images down to a depth of 5 m. The dashed boxes interpreted as platy rocks/bedding rocks. As an example, the P1 box shows the positions of the cm-scale quad-polarized images shown in Fig. 3.

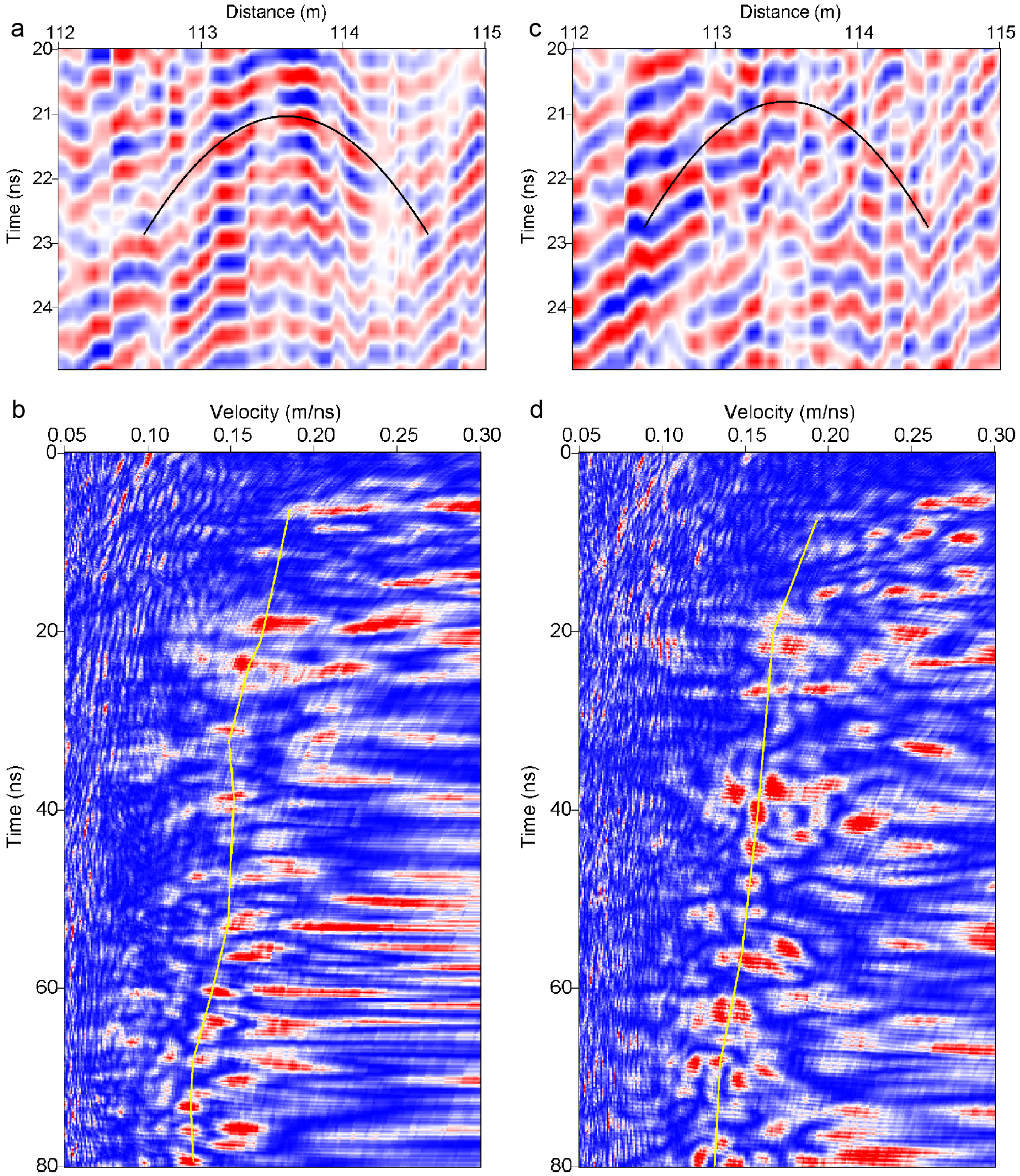

Figure S6. Diffracted velocity analysis for the HH and HV data. a, HH diffracted wave, where a solid yellow line indicates the hyperbolic trajectories of the diffracted wave curve, where the

peak of the hyperbolic curve is x = 113.6 m and t = 21.0305 ns. **b**, Velocity spectrum of **a**. **c**, HV-diffracted wave corresponding to the peak point of the hyperbolic curve at x = 113.5 m and t = 20.811. **d**, Velocity spectrum of **c**.

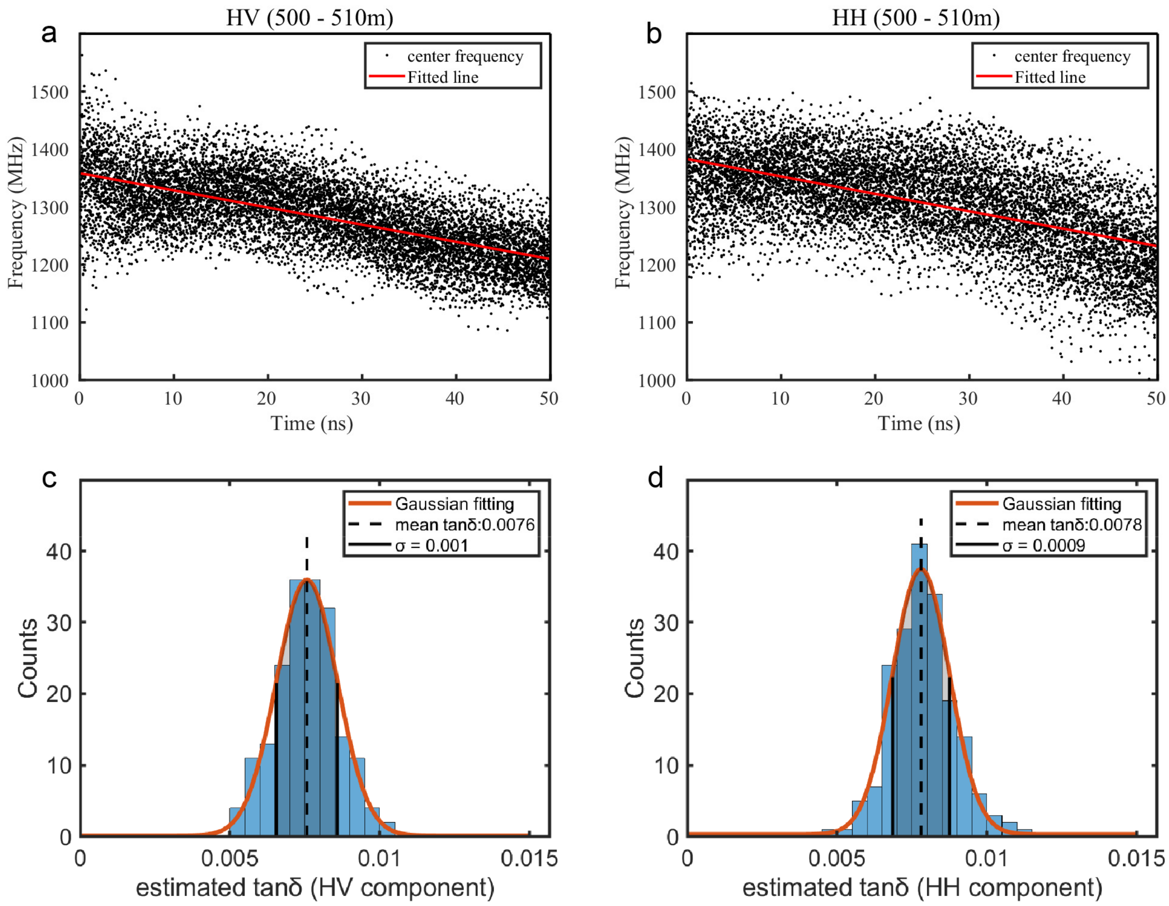

Figure S7. Loss tangent estimated via the centroid frequency-shift method. Panels **a** and **b** show the centroid frequency versus time for the HH and HV components from distances ranging from 500 to 510 m (approximately 1000 traces). The black dots represent the centroid frequency at different times, whereas the red lines are the fitting results. The horizontal axis represents the two-way traveltime (ns), and the vertical axis represents the centroid frequency (MHz). Panels **a** and **b** show histograms of the loss tangent estimated by the HH and HV components. The loss tangents are 0.0076 for HV and 0.0078 for HH.
